# Effect of natural and synthetic noise data augmentation on physical action classification by brain–computer interface and deep learning

**DOI:** 10.3389/fninf.2025.1521805

**Published:** 2025-02-27

**Authors:** Yuri Gordienko, Nikita Gordienko, Vladyslav Taran, Anis Rojbi, Sergii Telenyk, Sergii Stirenko

**Affiliations:** ^1^Computer Engineering Department, National Technical University of Ukraine “Igor Sikorsky Kyiv Polytechnic Institute,” Kyiv, Ukraine; ^2^Laboratoire Cognitions Humaine et Artificielle, Université Paris 8, Paris, France; ^3^Department of Automation and Computer Science, Faculty of Electrical and Computer Engineering, Cracow University of Technology, Cracow, Poland

**Keywords:** deep neural network, brain-computer interface, grasp-and-lift, physical action, data augmentation, noise, noise data augmentation, detrended fluctuation analysis

## Abstract

Analysis of electroencephalography (EEG) signals gathered by brain–computer interface (BCI) recently demonstrated that deep neural networks (DNNs) can be effectively used for investigation of time sequences for physical actions (PA) classification. In this study, the relatively simple DNN with fully connected network (FCN) components and convolutional neural network (CNN) components was considered to classify finger-palm-hand manipulations each from the grasp-and-lift (GAL) dataset. The main aim of this study was to imitate and investigate environmental influence by the proposed noise data augmentation (NDA) of two kinds: (i) natural NDA by inclusion of noise EEG data from neighboring regions by increasing the sampling size *N* and the different offset values for sample labeling and (ii) synthetic NDA by adding the generated Gaussian noise. The natural NDA by increasing *N* leads to the higher micro and macro area under the curve (AUC) for receiver operating curve values for the bigger *N* values than usage of synthetic NDA. The detrended fluctuation analysis (DFA) was applied to investigate the fluctuation properties and calculate the correspondent Hurst exponents *H* for the quantitative characterization of the fluctuation variability. *H* values for the low time window scales (< 2 s) are higher in comparison with ones for the bigger time window scales. For example, *H* more than 2–3 times higher for some PAs, i.e., it means that the shorter EEG fragments (< 2 s) demonstrate the scaling behavior of the higher complexity than the longer fragments. As far as these results were obtained by the relatively small DNN with the low resource requirements, this approach can be promising for porting such models to Edge Computing infrastructures on devices with the very limited computational resources.

## 1 Introduction

Recently, deep learning (DL) methods based on deep neural networks (DNNs) were effectively used for processing different data (LeCun et al., 2015). In healthcare and elderly care, they become very popular for processing the very complex multimodal medical data (Chen and Jain, 2020; Esteva et al., 2019). Usage of DL is especially important in the view of availability of various brain–computer interfaces (BCI) used for collection and analysis of electroencephalography (EEG) signals generated by brain activities (Roy et al., 2019; Kotowski et al., 2020; Lawhern et al., 2018). In the context of critically important tasks, for example, for air-space applications, BCIs are intensively used for the mental workload assessment on professional air traffic controllers during realistic air traffic control tasks (Aricò et al., 2016b,a; Di Flumeri et al., 2019).

DNNs were actively used for analysis of EEG data in a different fields (Li et al., 2020; Aggarwal and Chugh, 2022; Zabcikova et al., 2022) such as air-space (Aricò et al., 2016b,a; Di Flumeri et al., 2019), medicine (Chen et al., 2022; Wan et al., 2019; Gu et al., 2021), education (Xu and Zhong, 2018; Gang et al., 2018; Belo et al., 2021), entertainment (Kerous et al., 2018; Gang et al., 2018; Vasiljevic and de Miranda, 2020; Cattan, 2021), and other applications (Zabcikova et al., 2022). Usually, components of convolutional neural network (CNN) (Lawhern et al., 2018; Lin et al., 2020; Gu et al., 2021; Gatti et al., 2019; Gordienko et al., 2021c), recurrent neural networks (RNN) (An and Cho, 2016; Wang et al., 2018b; Pancholi et al., 2021; Kostiukevych et al., 2021), and other including components of fully connected networks (FCN) (Gordienko et al., 2021c; Kostiukevych et al., 2021) are used in them. These models combine some methods of EEG feature extraction with the use of various filters and show significant improvement of performance in comparison with other models. For instance, 3D CNN model based on multi-dimensional feature combination improves the classification accuracy of sensorimotor area activated tasks in the brain (Wei and Lin, 2020). Some of the DNN models demonstrated their quite high efficiency on some tasks such as sleep stage classification, stress recognition, fatigue detection, motor imagery classification, emotion recognition, and emotion classification (Gu et al., 2021). As to the domain operator-specific scenarios, some interesting results were obtained for EEG hand movement force and speed forecasting with the accuracy >80% (Gatti et al., 2019) and the conflict prediction accuracy ≈60% (Vahid et al., 2020).

Some hybridization approaches become popular recently. For example, CNN components combined with RNN components (including long short-term memory (LSTM) blocks) were investigated recently to resolve action classification problem. For instance, various RNN architectures were compared in performance for identifying hand motions for GAL dataset from EEG recordings (An and Cho, 2016; Kostiukevych et al., 2021) and for AJILE dataset (Wang et al., 2018b).

As it is well-known in computer vision, for example, for image classification tasks, data augmentation (DA), in general, and noise data augmentation (NDA), in part, can improve the performance of DNNs. Various strategies for applying DA methods to EEG data were considered recently that allow to improve classification accuracy when the limited volume of the data is available (George et al., 2022). NDA methods can be performed by adding Gaussian noise (Cecotti et al., 2015; Freer and Yang, 2020; Gordienko et al., 2021c) or by creating synthetic EEG data (Zhang and Liu, 2018; Aznan et al., 2019; Fahimi et al., 2020). The similar numerous NDA-related approaches were proposed (Freer and Yang, 2020; Gordienko et al., 2021c; George et al., 2022), and many others were reviewed recently in several surveys (Rommel et al., 2022; Lashgari et al., 2020; Talavera et al., 2022).

Although the results are promising and intriguing, their statistical reliability remains uncertain due to potential external influences under real-world conditions. That is why the main of this study was to imitate and investigate environmental influence by the proposed NDA of two kinds: (i) natural NDA by inclusion of noise EEG data from neighboring regions by increasing the sampling size *N* and the different offset values for sample labeling (see details below) and (ii) synthetic NDA by adding the generated Gaussian noise.

It should be noted that DA is a widely used technique that enhances a model's ability to generalize by making it more robust to variations in input data. Common DA methods include geometric transformations, noise-based modifications (such as roughening, adding, or mixing), and generative approaches. However, in EEG analysis, geometric transformations such as scaling, rotation, and reflection are not directly applicable. Unlike structured tables, text, or images, EEG signals are continuous and vary over time. Even after feature extraction, they remain time series data. Applying geometric transformations, such as rotation, to EEG signals would disrupt their temporal structure, compromising their meaningful features.

Among various ways for adding noise to the EEG signals in purpose of DA Li et al. (2019); Parvan et al. (2019); Ko et al. (2021); Sun and Mou (2023), the following are of great interest due to their intuitive understanding:

- inject various types of noise (such as uniform, Gaussian, Poisson, salt, and pepper noise, and various color noise types) with different parameters (for instance: mean and standard deviation).
- manipulate the time segment of interest by shifting/adding/cropping/combining operations with including/subtracting the information about background and signal.
- synthesize the signal by encoding/decoding and generative approaches.

Like geometric transformation methods, noise addition-based DA has been widely applied in successful DL studies for CV (Simonyan and Zisserman, 2014; He et al., 2016). This approach enhances DA by introducing randomly sampled noise values into the original data. Injecting structured noise patterns (e.g., white Gaussian or pink noise) with a specific signal-to-noise ratio (SNR) can alter the spectral characteristics of a time series by introducing additional frequency components to the signal spectrum (Borra et al., 2024).

In the context of DA for EEG, numerous studies were performed to investigate the impact the noise-induced DA for EEG. Some of the recent results are shortly summarized in Table 1.

**Table 1:** Examples of EEG classification studies (“Reference” column) with noise-based DA with various noise parameters (“Noise Type”) on some standard or custom datasets (“Dataset Reference”) with the different numbers of classes (“*N*_*c*_”), neural network architectures (“NNA”), and the reported improved accuracy (“Accuracy, (%)”) by absolute values or changes (denoted with + sign).

**References**	**Noise**	**Dataset**	***N*_*c*_**	**NNA**	**Accuracy (%)**
**Gaussian White Noise**					
Zhang et al., 2017	NA	custom (Zhang et al., 2017)	4	CNN	97.5
Behncke et al., 2018	NA	KPO (Behncke et al., 2018)	2	ConvNet	75 ± 9
Behncke et al., 2018	NA	RGO (Behncke et al., 2018)	2	ConvNet	62 ± 7
Lashgari et al., 2021	*N*(0, *set*)	BCI C 2008 2a (Brunner et al., 2008)	4	CNNwA	93.6 ≥ 91.57
Lashgari et al., 2021	*N*(0, *set*)	BCI C 2008 2b (Leeb et al., 2008)	2	CNNwA	87.83 ≥ 87.60
George et al., 2022	*N*(0, *c*_*mean*_)	custom (Cho et al., 2017b)	4	CNN	78.30-86.51 ≥ 77.73
George et al., 2022	*N*(0, *c*_*mean*_)	EEG-BCI (Kaya et al., 2018)	2	CNN	81.74-83.01 ≥ 80.73
Tunnell et al., 2022	*N*(0, 1)	DEAP (Koelstra et al., 2011)	2	EEGNet	77.16 ≥ 66.30
Wu et al., 2022	*N*(0, *set*)	SleepEDF (Kemp et al., 2000)	5	neuro2vec	86.53 ≥ 85.49
Wu et al., 2022	*N*(0, *set*)	Epilepsy (Andrzejak et al., 2001)	2	neuro2vec	44.30 ≥ 40.24
Wu et al., 2022	*N*(0, *set*)	Ninapro (Pizzolato et al., 2017)	18/40	neuro2vec	86.69 ≥ 84.32
Gou et al., 2022	NA	BCI-CRC-WRC (Pizzolato et al., 2017)	3	EEGNet	54.72 ≥ 48.34
Collazos-Huertas et al., 2023	NA	GigaScience (Cho et al., 2017a)	10	EEGNet+ScoreCam	78.2 ≥ 69.7
Lopez et al., 2023	*N*(0, *MW*)	MAHNOB-HCI (Soleymani et al., 2011)	3(a)	HyperFuseNet	41.56 ≥ 40.90
Lopez et al., 2023	*N*(0, *MW*)	MAHNOB-HCI (Soleymani et al., 2011)	3(v)	HyperFuseNet	44.30 ≥ 40.24
Ashfaq et al., 2024	*N*(0, 0.01)	CogAge (Nisar et al., 2020)	7	MHyCoL	+5, +30
Ashfaq et al., 2024	*N*(0, 0.01)	UniMiB-SHAR Micucci et al., 2017	17	MHyCoL	+5, +30
Lee et al., 2024	NA	BCI C 2020 (Jeong et al., 2022)	6	DeiT	+0.49 … 10.57
Cai et al., 2024	*N*(*set, set*)	custom (Cai et al., 2024)	6	CNN	85.2 ≥ 58.6
Wang et al., 2024	NA	OpenBMI (Lee et al., 2019)	2	MRCNN	82.47 ≥ 79.45
Wang et al., 2024	NA	SingleArmMI (Wang et al., 2024)	4	MRCNN	43.19 ≥ 37.36
Falaschetti et al., 2024	*N*(0, 0.03)	custom (Falaschetti et al., 2024)	6	LSTM	95.2
Borra et al., 2024	NA	9 MOABB sets (Jayaram and Barachant, 2018)	2/4	SpeechBrain-MOABB	+14… 25.2
Cho et al., 2017b	NA	custom (Cho et al., 2017b)	3	CNN	93–95
Ouyang et al., 2024	NA	custom Brainlink (Ouyang et al., 2024)	4	BRIEDGE	98.78 ≥ 98.07
Ouyang et al., 2024	NA	custom EyeState (Ouyang et al., 2024)	2	BRIEDGE	92.51 ≥ 84.75
Ouyang et al., 2024	NA	custom BCI-2000 (Ouyang et al., 2024)	4	BRIEDGE	66.02 ≥ 56.05
Ouyang et al., 2024	NA	custom Hybrid (Ouyang et al., 2024)	10	BRIEDGE	86.50 ≥ 63.72
**Uniform noise**					
Freer and Yang, 2020	[–0.5,5] scaled	BCI C IV (Tangermann et al., 2012)	4	CNN	+5.3
**Various noise types (uniform, white, pink, impulse)**					
Sun et al., 2024	NA	Multi-dataset (Sun et al., 2024)	3	CNN+GCN+Transf	96.7,93.3,93.3

[1] NA, the noise parameters were not detailed (not available) in the studies.

[2] *set*, the set of various values was investigated.

[3] *c*_*mean*_, the Gaussian noise is generated with zero mean and standard deviation equal to the class mean.

[4] *MW*, the Gaussian noise signal with zero mean is added to each sample, with its standard deviation being computed modality-wise (MW).

[5] (a), (v), two classification schemes: (a) - the arousal scheme (3 classes: calm, medium aroused, and excited), (v) - the valence scheme (3 classes: unpleasant, neutral valence, and pleasant).

DA by Gaussian noise involves adding Gaussian white noise to recorded EEG signals (Wang et al., 2018a). In practice, a perturbation *E*(*t*)~*N*(0, σ^2^) is independently sampled for each channel and acquisition time and added to the original signal *X*, resulting in the augmented data: [*X*](*t*) = *X*(*t*)+*E*(*t*) Here, σ represents the standard deviation of the noise distribution. This parameter determines the magnitude of the transformation as larger values lead to greater distortion of the original signal. The primary motivation for this DA is to enhance model robustness against noise in EEG recordings, which are known to have a limited SNR. Gaussian noise augmentation is controlled by the parameter σ, which dictates the standard deviation of the sampled noise. Selecting an appropriate σ value is as crucial as choosing the DA method itself. For example, when σ exceeds 0.2, EEG signals become excessively noisy, making the DA systematically detrimental to learning (Rommel et al., 2022).

For instance, spectrogram images of motor imagery EEG have been augmented by introducing Gaussian noise (Zhang et al., 2020). White noise manifests as random fluctuations uniformly distributed across all frequencies in the EEG signal. It can originate from various sources, including thermal noise in EEG equipment, sensor artifacts, or external electrical interference. This noise reduces the SNR, making it challenging to discern neural patterns and potentially masking true neural activity. The effect is especially problematic for low-amplitude signals, such as those originating from deep brain regions.

Another method for increasing data diversity involves injecting random matrices into the raw data, typically sampled from Gaussian distributions (Okafor et al., 2017). Gaussian noise injection applies a randomly generated matrix from a Gaussian distribution to the original data as a form of DA. While these methods are straightforward and intuitive, they can sometimes exacerbate model overfitting due to the high similarity between the original and augmented data. In particular, several studies have augmented EMG signals by adding Gaussian noise to the original dataset and adjusting the SNR (Atzori et al., 2016; Zhengyi et al., 2017; Tsinganos et al., 2018).

Another study uses injecting random Gaussian noise generated based on the statistical properties of the data. The mean value of trials for the target class is computed; then, Gaussian noise with a zero mean and a standard deviation equal to the class mean (*c*_*mean*_ in Table 1) is generated. This noise is added to randomly selected trials to create artificial frames. This simple yet effective method preserves the original waveform characteristics while introducing slight numerical variations across trials (George et al., 2022).

In other studies, DA has proven effective in addressing the challenge of limited learning caused by small training sets in EEGNet, leading to significant improvements in classification accuracy. As a result, the data were expanded by a factor of three, and the standard deviation of the added Gaussian noise was set to 0.1 (Cai et al., 2024).

The modality-wise approach was proposed in Lopez et al. (2023), where the Gaussian noise signal with zero mean is added to each sample, with its standard deviation computed modality-wise (*MW* in Table 1), ensuring that the augmented signal achieves a signal-to-noise ratio (SNR) of 5dB.

In addition to Gaussian noise, various types of colored noise can also be present in EEG signals due to physiological and environmental factors. These noise types typically manifest as interference, distorting the true brain activity and complicating accurate analysis and interpretation. Below are some examples of how different types of colored noise may appear in EEG signals of brain activity:

Pink noise (1/f noise) is characterized by greater power at lower frequencies, with a gradual decrease in power as the frequency increases. This type of noise can naturally arise from brain activity, particularly during resting states, or be introduced by background physiological processes such as muscle activity or skin potentials. Pink noise can dominate low-frequency bands, potentially masking slow-wave oscillations that are crucial for sleep studies or resting-state EEG analysis.

Brown (red) noise displays even more power at lower frequencies than pink noise, with a steeper decline as the frequency increases. It can arise from long-term drift in electrode potentials or baseline shifts in the EEG signal, often caused by environmental factors that affect the EEG setup. This results in large, slow oscillations that can dominate the EEG trace, potentially masking lower-frequency brain rhythms. While Gaussian (white) noise is frequently applied, the specific use of brown noise has not been widely explored in the literature, but exploring brown noise injection could potentially offer new avenues for enhancing EEG data augmentation techniques.

Blue noise is characterized by an emphasis on high frequencies, manifesting as rapid, small-amplitude fluctuations in the signal. It can originate from high-frequency environmental interference, such as electronic devices or power lines, or from muscle activity, including micro-movements of the scalp or jaw. This type of noise can mask high-frequency neural signals, such as gamma rhythms (30–100 Hz), and may lead to false-positive detections in high-frequency analyses.

Violet noise is an extreme form of high-frequency noise, with a stronger emphasis on higher frequencies than blue noise. It can be caused by electronic interference within the EEG system, such as sudden changes in electrode contact, such as detachment or movement. This noise can introduce sharp spikes or rapid fluctuations that resemble artifacts, potentially disrupting the analysis of high-frequency components, such as event-related potentials (ERPs).

In some studies, several types of noises (in addition to white Gaussian noise) were investigated (Tangermann et al., 2012; Sun et al., 2024). To increase the number of training samples and address the variability and randomness of EEG signals, several noise DA strategies were implemented (Sun et al., 2024). Specifically, the noise DA strategies were adopted to enhance EEG data by simulating various noise sources that may be encountered in real-world environments. The types of noise applied were: (1) uniform noise, (2) Gaussian noise, (3) pink noise, (4) impulse noise, and (5) power-line noise. These noise types were randomly incorporated into the processed clean EEG signals at different proportions (ranging from 10% to 70% of the average amplitude of the EEG signal), thus generating a greater number of training samples. These DA strategies not only enhance the model's robustness to existing noise in the original signals but also improve the model's generalization capabilities in the presence of unknown noises. The introduction of noise through DA strategies has a positive effect on model training, particularly with Gaussian and pink noise. This suggests that such disturbances are prevalent in real EEG data as the noise-augmented strategies enhance the diversity of the samples and the generalization ability of the model. Overall, as the intensity of the added noise increases, both pink noise and Gaussian noise initially decrease and then increase the model prediction error. The optimal results are achieved when noise is added at 30% of the average signal intensity.

In the field of DA for EEG, the use of random shifts has been explored to some extent. However, dataset shift (where the data distribution during inference differs from that during training) is common in real biosignal-based applications. To enhance robustness, probabilistic models with uncertainty quantification are adapted to assess the reliability of predictions. Despite this, evaluating the quality of the estimated uncertainty remains a challenge. Recently, the framework was proposed to assess the ability of estimated uncertainty to capture various types of biosignal dataset shifts with different magnitudes (Xia et al., 2022). Specifically, three classification tasks were used that were based on respiratory sounds and electrocardiography signals to benchmark five representative uncertainty quantification methods. Extensive experiments reveal that, while Ensemble and Bayesian models provide relatively better uncertainty estimates under dataset shifts, all the tested models fall short in offering trustworthy predictions and proper model calibration. In another study, time-axis shifts of EEG trials were applied to generate artificial signals for DA purposes (Sakai et al., 2017). Again, the effectiveness of such geometric transformations is debated. Given the non-stationary nature of EEG signals, transformations like shifting may interfere with inherent features, potentially corrupting the data (Kalashami et al., 2022). Thus, while random shifts have been used in DA for EEG, their effectiveness continues to be a subject of ongoing research and discussion.

Another approach to applying DA for EEG involves noise injection through the inclusion of neighboring regions and other manipulations with data. A recent study utilized such DA strategies to address the challenge of small sample sizes. Specifically, translations and vertical flip operations were employed to capture a broader range of temporal information. The data were extracted from 0 to 500 ms after stimulation and then translated. Five time points within the first 200 ms after stimulus onset were randomly selected, and data from 500 ms later were collected. This method increased the dataset size 6-fold. Subsequently, the data were flipped by taking the opposite value of the augmented data, further expanding the dataset to 12 times its original size (Gou et al., 2022).

Similarly, other research efforts explore different data augmentation strategies, including Generative Adversarial Networks (GANs) and Variational Autoencoders (VAEs), to generate synthetic EEG data for training (Habashi et al., 2023; Ibrahim et al., 2024).

GANs, initially introduced for image generation, have also shown promise as a potential DA solution for EEG. GANs and their variants generate artificial data by training two competing networks: a generative network and a discriminative network. The generative network takes random noise from a predefined distribution (e.g., Gaussian) and attempts to create synthetic data that resemble real samples, while the discriminative network is trained to differentiate between real and synthetic data. Through adversarial training, the generative network progressively improves, ultimately producing highly realistic EEG signals (Zhang et al., 2021; Bao et al., 2021; Carrle et al., 2023; Ibrahim et al., 2024).

VAEs offer another approach to generating synthetic EEG data. Like a conventional autoencoder, a VAE consists of an encoder that transforms raw data into a latent representation and a decoder that reconstructs the data from this latent space. To generate new samples, the VAE randomly samples points from the learned latent distribution and passes them through the decoder, which reconstructs them into novel data. Both GANs and VAEs generate new samples indirectly by learning meaningful latent representations of the original data (Bao et al., 2021; Sun and Mou, 2023).

The proposed study contributes to investigation of novel approaches for noise-based DA for EEG classification with emphasis on influence of adding the randomly generated artificial noise and the natural noise created by inclusion of neighboring EEG data segments. This exploration is crucial as it could reveal and compare how artificial and natural noise DA can impact EEG classification performance for various noise DA parameters, for example, with increased sample size, varied offsets, etc. It is especially important for lightweight DNN architectures, designed for Edge Intelligence setups, to ensure efficient EEG processing with minimal computational resources, advancing biologically relevant and computationally efficient DA methods.

For effective application of noise-based DA methods, a clear understanding of the characteristics and sources of these noise types in real-world scenarios is necessary. That is why this study is limited to the simplest noise types that can be intuitively understandable and potentially interpreted. This study aims to provide a thorough understanding of noise-based DA by Gaussian noise injection to mimic random fluctuations evenly distributed across all frequencies in the EEG signal that can be caused by the environment.

The methods of statistical analysis and detrended fluctuation analysis (DFA) are widely used to investigate the fluctuation properties of the measured metrics and calculate the correspondent Hurst exponents (Hurst, 1956). For this purpose, the relatively small DNN [that was described and analyzed in details in Gordienko et al. (2021c)] with components of FCNs and CNNs was considered to classify physical activities (namely, hand manipulations) from the grasp-and-lift (GAL) dataset (Luciw et al., 2014; Kaggle, 2020). The special attention was paid to the analysis of the previous, mid- and post-action segments of the corresponding brain activity to anticipate them before the start of the action.

Finally, this study is targeted on investigation of EEG data collected by BCI to resolve classification problem for some physical activities (namely, hand manipulations) by the relatively simple DNN. The DNN was applied for analysis of preliminary (prior-activity), current (in-activity), and following (post-activity) parts of the relevant brain EEG signals. This problem is very important in the view of complex practical conditions where EEG activity can be disturbed by other physiological activities and, especially, external environmental noise. On the one hand, such disturbances can worsen the classification performance but, on the other hand, in reverse can improve it if it will be used during training as data augmentation (DA) technique.

## 2 Materials and methods

In this section, several important experimental aspects are explained: the dataset with EEG brain activities for six types of physical activities, structure of the model, metrics, workflow, and data augmentation techniques.

### 2.1 Dataset

**Figure 1:**
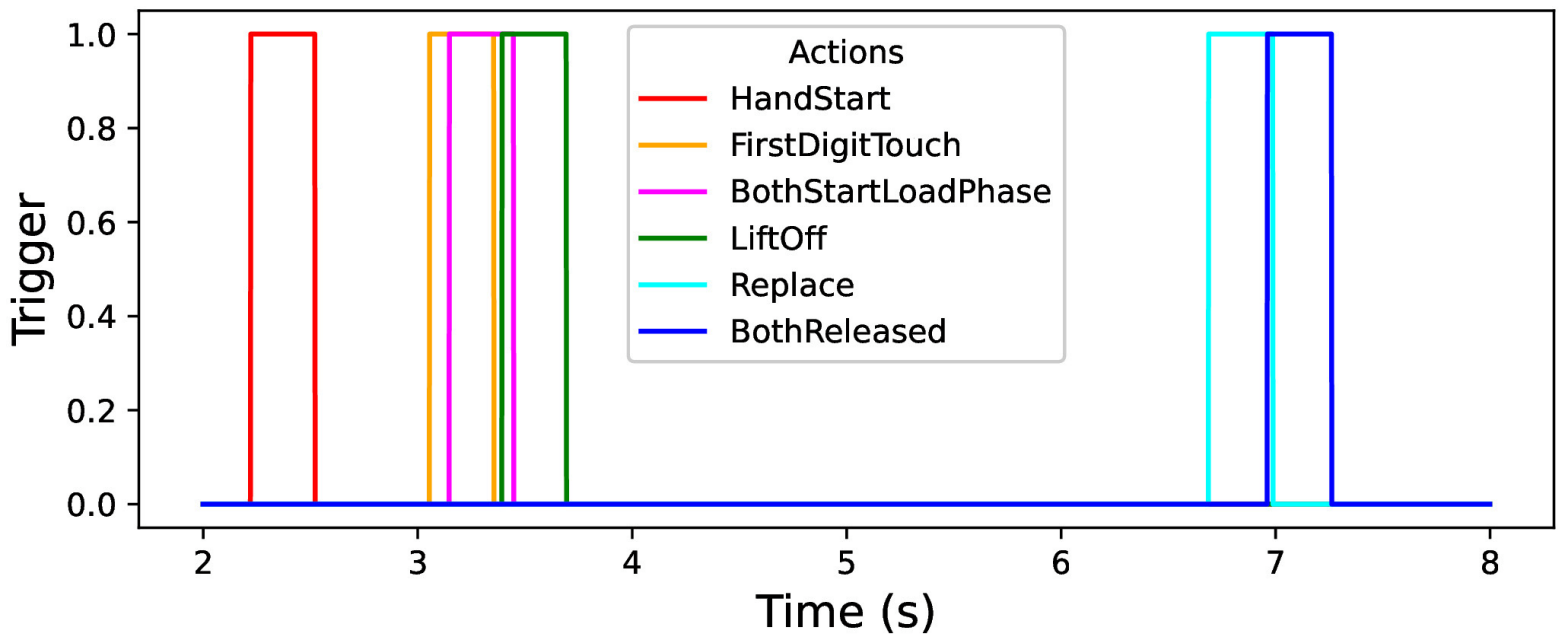
Timeline of physical actions: trigger channel vs. time.

In this study, the open “grasp-and-lift” (GAL) dataset is used that contains information about brain activity of 12 persons (Luciw et al., 2014; Kaggle, 2020): more than 3,900 trials (monitored and measured by the sampling rate of 500 Hz) in 32 channels of the recorded EEG signals. The person tries to perform six types of physical activities, namely: “HandStart”—moves hand to an object (for example, some gadget), “FirstDigitTouch”—touches the object by finger (for example, press a button), “BothStartLoadPhase”—takes (“grasps”) the object by fingers, LiftOff—raises (“lifts”) the object by fingers, Replace—returns the object by fingers back, BothReleased–releases fingers. The data from GAL dataset were previously processed in a standard way (Kostiukevych et al., 2021; Gordienko et al., 2021c) with taking into account the correspondent time position of physical actions (actually hand movements here) and their duration (Figure 1).

It should be emphasized that these kinds of physical activity can be naively divided in three parts depending on the feasibility of their classification: the easiest (HandStart), medium (LiftOff, Replace, and BothReleased), and hardest (BothStartLoadPhase and FirstDigitTouch) classification. But BothStartLoadPhase and FirstDigitTouch activities strongly overlap in this experiment and that is why hardly can be recognized as separate activities (this is planned to be fixed by collection of the original data in the same fashion in our future research).

**Figure 2:**
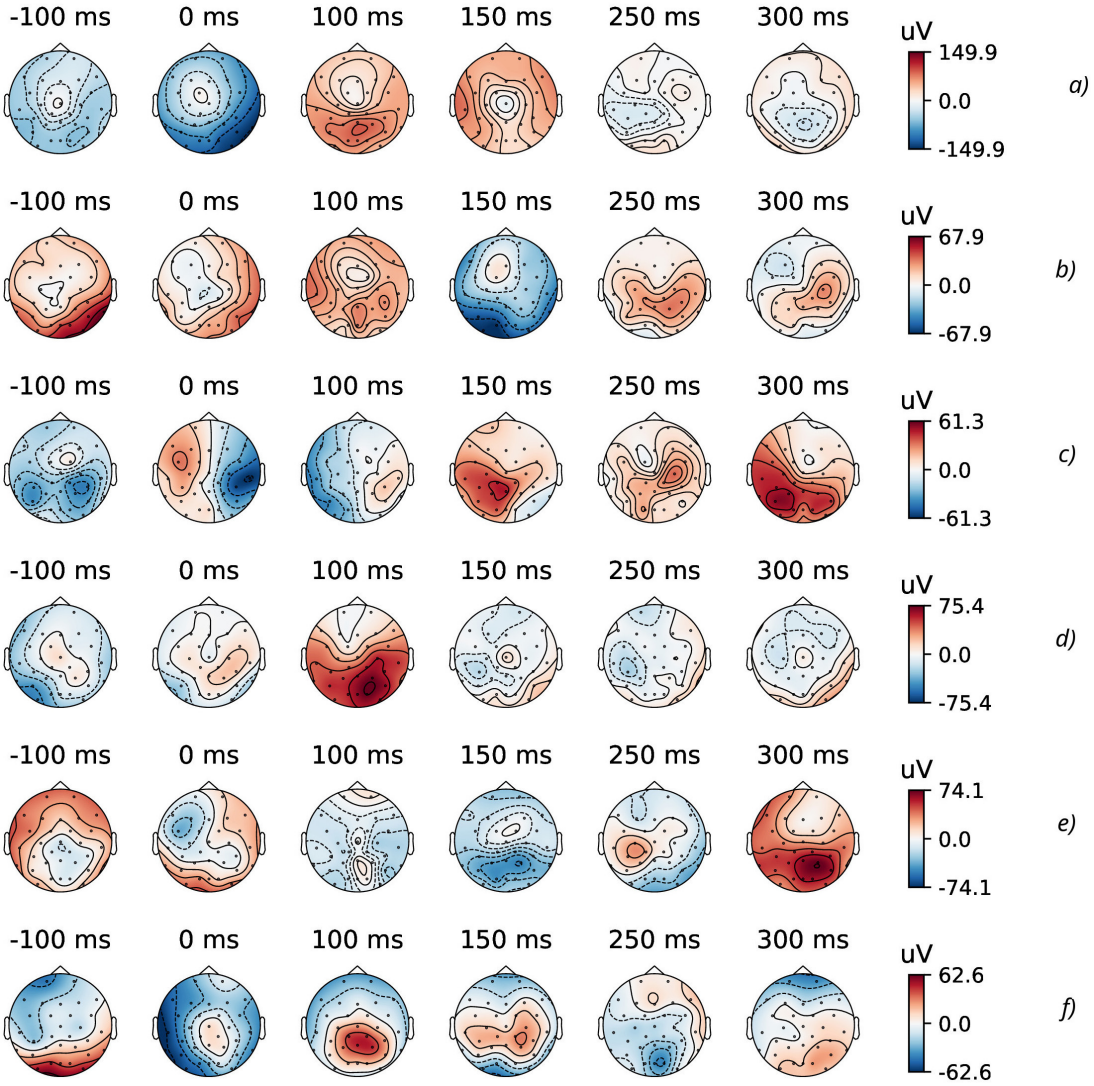
Topographic maps of specific time points of evoked data (from 32 EEG channels) for the considered physical actions: **(A)** HandStart, **(B)** FirstDigitTouch, **(C)** BothStartLoadPhase, **(D)** LiftOff, **(E)** Replace, and **(F)** BothReleased actions.

As a part of an explanatory data analysis (EDA), visualizing and analyzing the experimental EEG data from GAL dataset (Gramfort et al., 2013) was performed by means of MNE open-source Python (Gramfort et al., 2013). For example, all EEG data measured by the BCI-sensors with their predefined spatial position can be plotted as subtopomaps of an evoked potential trough timeline (Figure 2).

### 2.2 Models

From subtopomaps of an evoked potential trough timeline (Figure 2), one can evidently see the complex distribution of EEG sifgnals over scalp. As far as EEG signals interfere with each other due to their electromagnetic nature (Figure 2), the combinations of the data channels can be effectively used for their processing on the basis of FCN, CNN, and RNN like it was demonstrated in our previous studies (Gordienko et al., 2021c; Kostiukevych et al., 2021). In this research, the relatively small “vanilla” DNN (Gordienko et al., 2021c) was used here. The main motive for the usage of CNNs was to use convolution operations inside an EEG time sequence of each EEG channel where all 32 EEG channels were considered to be independent ones. Then, the workflows from 32 EEG channels were combined in fully connected dense layers and then transmitted to the classification layer. The idea is to use 1D convolution operations across all 32 EEG channels for each time step. The mentioned “vanilla” DNN contains three convolutional layers [with 32 filters and kernel (3,1); 64 filters and kernel (5,1); 128 filters and kernel (7,1)] followed by batch normalization and max pooling layers with pool kernel (2,1) with dropout (0.1) and FCN layers.

### 2.3 Metrics

Several standard metrics were used such as accuracy and loss that were calculated during validation phase of the model learning with checkpointing states for the minimal value and maximal value of loss and accuracy, respectively. In addition, the area under curve (AUC) was measured for receiver operating characteristic (ROC) with their micro and macro versions, and their mean and standard deviation values. It is important because for the given threshold, the accuracy measures the percentage of objects correctly classified, regardless of which class they belong to. As far as AUC is threshold-invariant, AUC can allow us to measure the quality of the models considered here independently from the selected classification threshold. The AUC can consider various possible thresholds and can provide the wider range of the classifier performance. During validation phase, the models with the best accuracy and loss values were saved for the testing phase. For smooth line fitting by locally weighted polynomial regression method (LOWESS) (Cleveland et al., 2017) using weighted least squares, giving more weight to points near the point whose response is being estimated and less weight to points further away.

To investigate the high level of the fluctuations of the measured metrics, that was observed in our previous studies, DFA (detrended fluctuation analysis) was applied here. DFA was proposed to study some memory effects in sequences of the complex biological structures (Peng et al., 1993). During the last decades, it was successfully used in investigation of sequences by means of the scaling properties of the fluctuation function *F*(*n*) of non-overlapping time intervals of length *n*. *F*(*n*) is expected to scale as *n*^*H*^, where *H* is the Hurst exponent (Hurst, 1956).

### 2.4 Workflow and data augmentation

**Figure 3:**
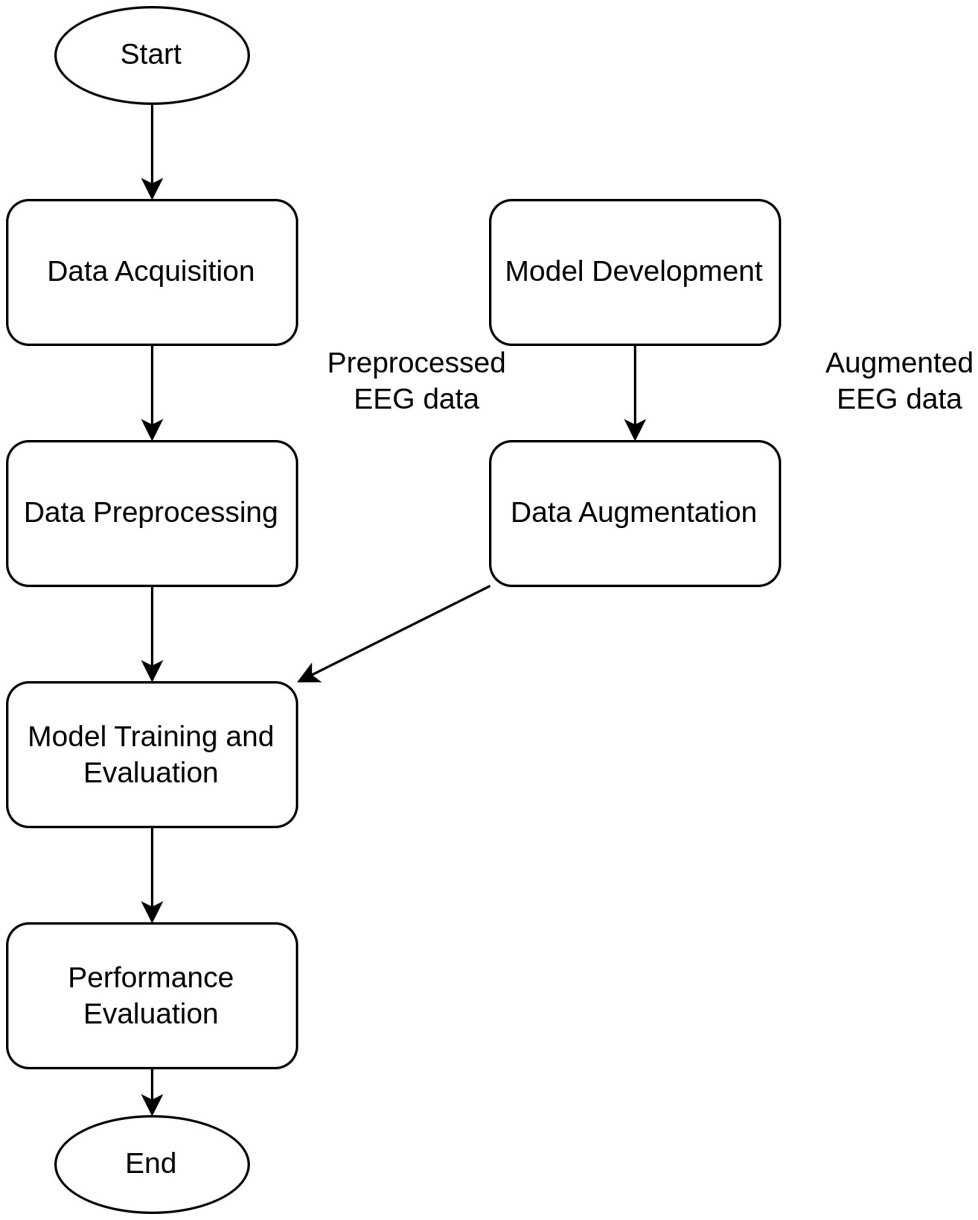
Main stages of the whole workflow.

The training, validation, and testing stages of the whole workflow (Figure 3) for the proposed simple DNN model were applied for the single epoch only, because the main aim was not the highest possible performance, but feasibility analysis of reliable classification under induced noise. The introduced noise was of two kinds: (i) natural NDA by inclusion of noise EEG data from neighboring regions by the different offset values (see details below) and (ii) synthetic NDA by adding the generated Gaussian noise.

During each training iteration, the callbacks were organized to save the best models (with the highest accuracy and lowest loss) for the subsequent testing stage. The number of signal samples (*N*) in each Input EEG time Sequence (IS) was in the range from 100 to 2,000. These ISs were collected in a random way from the whole timeline of the experimental EEG data.

To mimic the natural NDA, the labels of physical activities for each IS were defined by ground truth (GT) values in the following three locations: at the beginning, medium, and end moment inside IS. These positions were denoted by the offset values, for example, if offset = 0, then label (IS) = GT (beginning); if offset = 0.5, then label (IS) = GT (medium); if offset = 1, then label (IS) = GT (end). Actually, it allowed us to get the GT labeling without neighboring regions without the correspondent physical action (offset = 0.5), GT labeling with some neighboring regions after the correspondent physical action (offset = 0), and GT labeling with some neighboring regions before the correspondent physical action (offset = 1). Actually, GT labeling with offset = 0 and offset = 1 provides inclusion of some natural EEG noise after and before the actual physical action. Of course, EEG activity related to the physical action signal (PAS) can take place before and after the actual physical action, but the increase of the number of signal samples (*N*) can lead to the PAS-to-noise ratio (PAS-NR) decrease and imitate the higher influence of the natural noise.

Under these conditions, the training, validation, and testing stages were independently done in an iterative way for 20 values of *N* with the step of 100. Finally, *N* values were obtained in the range from 100 to 2,000 that resulted in 20 iterations of training, validation, and testing phases. For each instance of *N*, the dataset was distributed in approximate proportion of 82% (≈ 300 examples)/9% (≈ 300 examples)/9% (≈ 300 examples) for training/validation/testing sets, respectively. As a result, 20 trained models were obtained for each iteration (one model per each input sequence with *N* values); then, 20 sets of metrics, including AUC, and its micro and macro versions, were calculated and plots of these metrics vs. *N* were constructed (see below).

## 3 Experimental

### 3.1 DNN training/validation/testing stages

During EDA stage, the GAL data were preprocessed in the standard way described in details in our previous studies (Gordienko et al., 2021c; Kostiukevych et al., 2021). In Figure 2, the topographic maps of specific time points of evoked data (from 32 EEG channels) for some physical actions (FirstDigitTouch and LiftOff) are shown. Here, the most characteristic parts of EEG signals and their spatial distributions over a scalp are shown for the better understanding the very complex details of EEG brain activity.

As it was demonstrated before (Gordienko et al., 2021c; Kostiukevych et al., 2021), some physical actions (such as HandStart) are followed by very pronounced patterns with the local minimums and maximums, while many others (such as BothStartLoadPhase, LiftOff, Replace) are hardly recognizable by unique patterns. In addition, it should be noted for several actions (such as HandStart, and especially Replace and BothReleased) that significant brain activity is started some milliseconds before the correspondent movements, but it is quite dubious to make the same statement about other actions in the view of the unrecognizable different patterns. The main idea of this study is based on our previous studies (Gordienko et al., 2021c; Kostiukevych et al., 2021) and consists in the hypothesis that relatively small DNNs even can classify the EEG patterns of the currently undergoing physical actions in the presence of some induced noise even, but the additional aspects include the investigation of impact induced by natural and synthetic kinds of noise.

**Figure 4:**
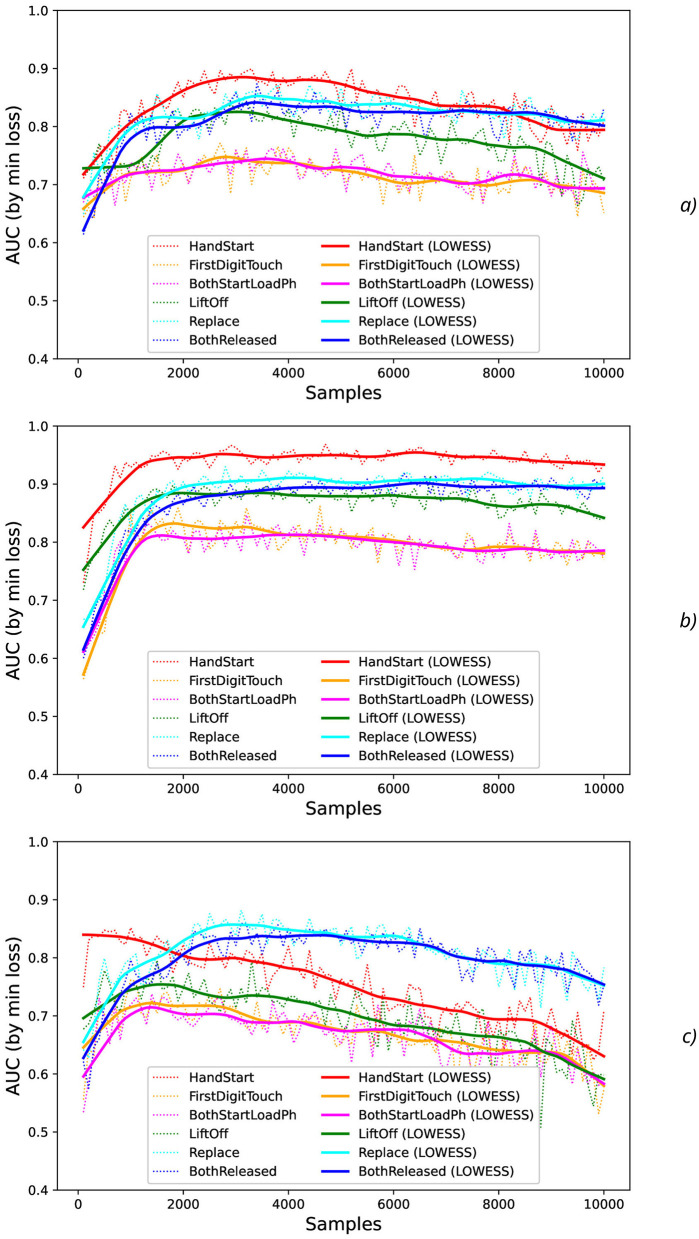
Comparison of AUC values as a function of the number of samples for the different physical actions (dotted lines) and their smoothed fits by LOWESS-method (solid lines) for the offsets imitating the natural noise addition: **(A)** 0, **(B)** 0.5, and **(C)** 1.

At testing stage, AUC values were measured (dotted lines in Figure 4) and their smoothed fits were obtained by LOWESS-method (solid lines in Figure 4). For various actions and offsets, AUC values (Figure 4) demonstrate the high intensity of fluctuations with increase of *N* that can be explained by the influence of the non-relevant (to the current physical activity) regions of the increased time sequence under investigation (imitating the natural noise addition).

### 3.2 Noise data augmentation

**Figure 5:**
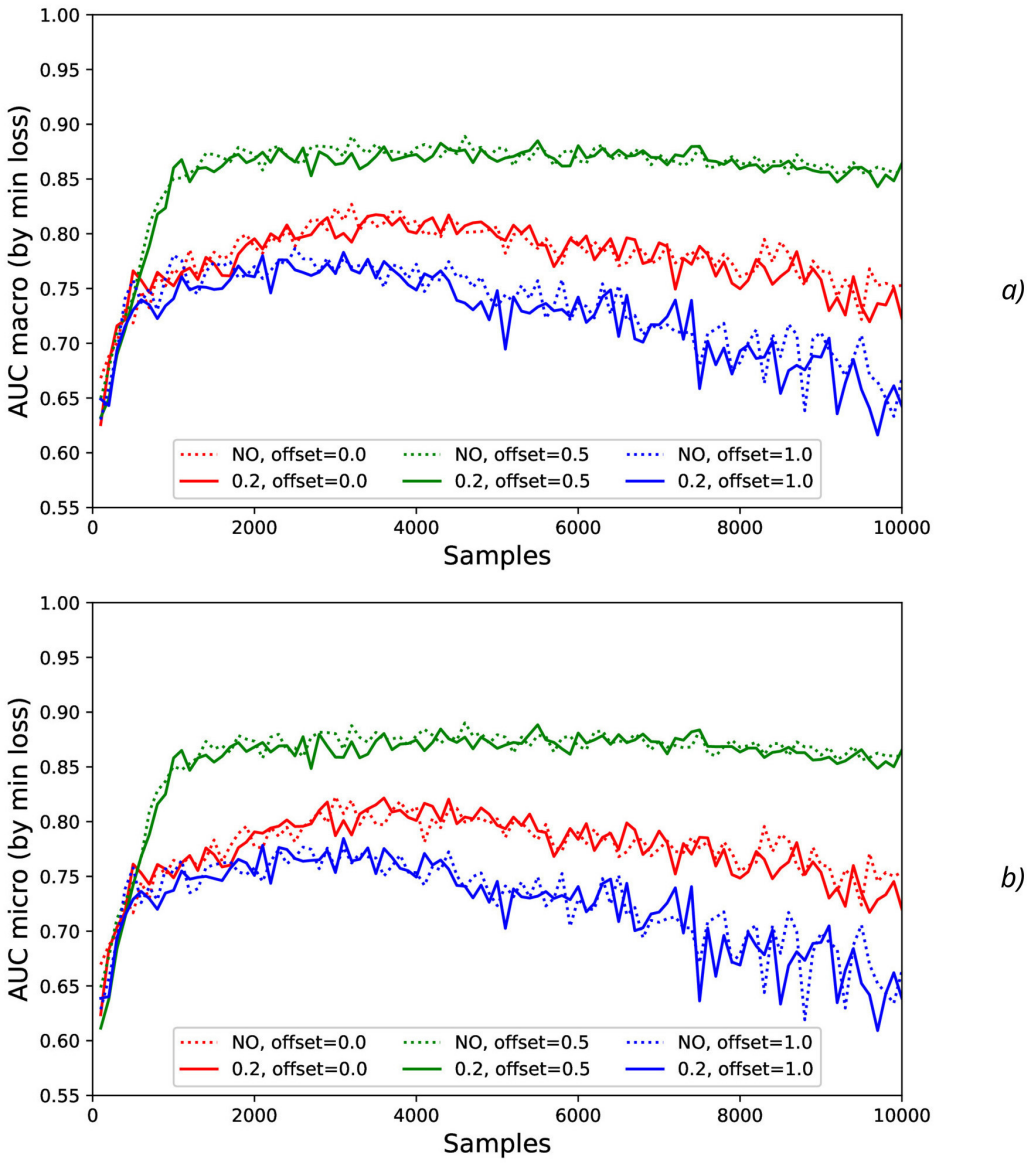
Comparison of macro **(A)** and micro **(B)** AUC values as a function of the number of samples for the different offset values (colors) without the synthetic noise (dotted lines) and with the synthetic noise σ_*synth*_ = 0.2 (solid lines).

The effect of the natural noise addition (by offsets 0 and 1 with various levels by increasing *N*) can be observed by calculation of the correspondent macro AUC (Figure 5A) and micro AUC (Figure 5B) values. It is evident that offsets 0 and 1 lead to the lower micro and macro AUC values in comparison with the GT labeling by offset = 0.5, and the AUC decrease is higher for the higher *N* values.

**Figure 6:**
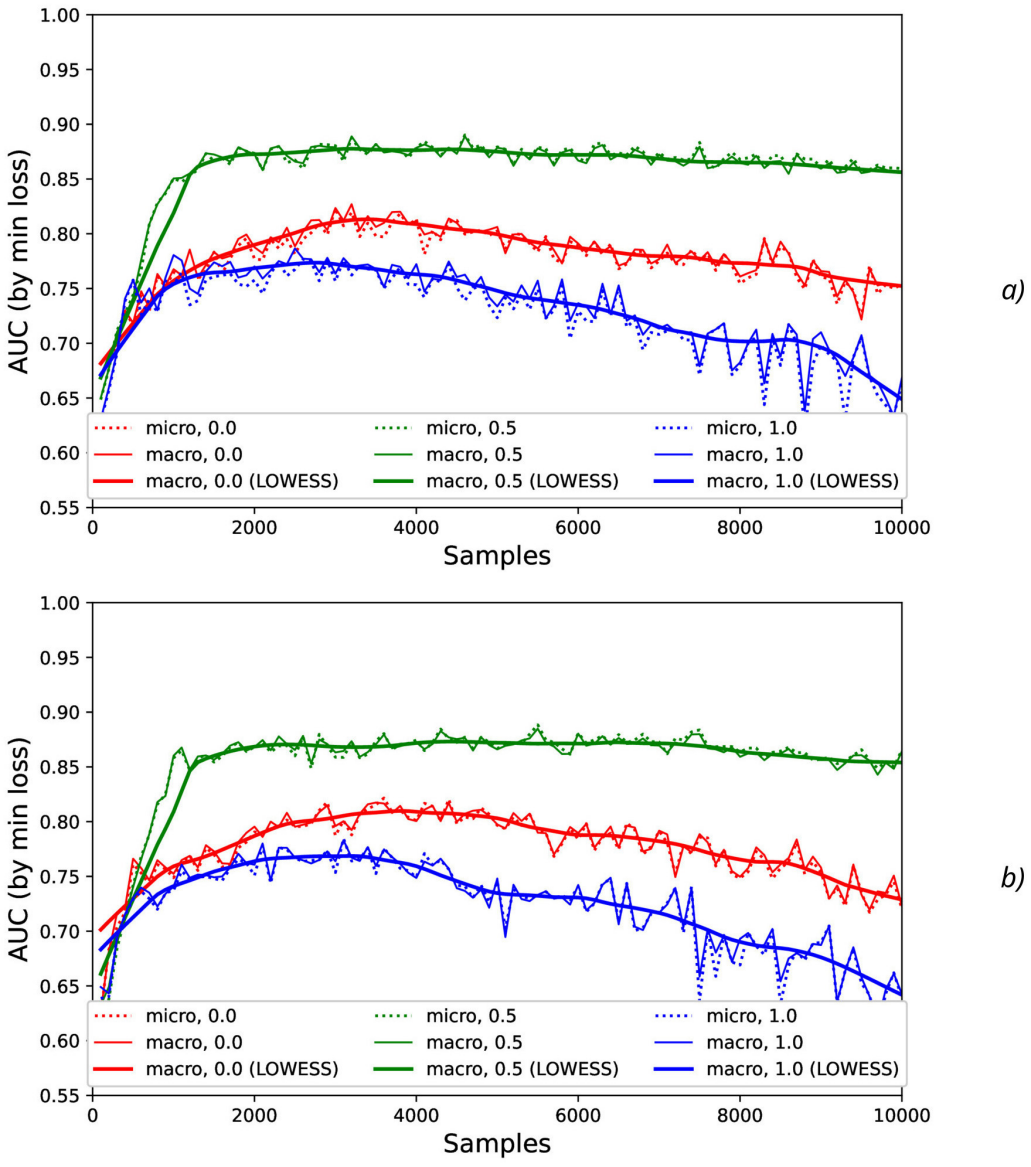
Comparison AUC values as a function of the number of samples for the different offset values (colors) without the synthetic noise **(A)** and with the synthetic noise σ_*synth*_ = 0.2 **(B)**.

To investigate stability of the results obtained, the additional artificial “synthetic” NDA as the values generated with mean = 0 and different standard deviations σ_*synth*_ (such as 0.001, 0.01, 0.1, and 0.2) was applied to the original normalized data (Figure 6).

**Figure 7:**
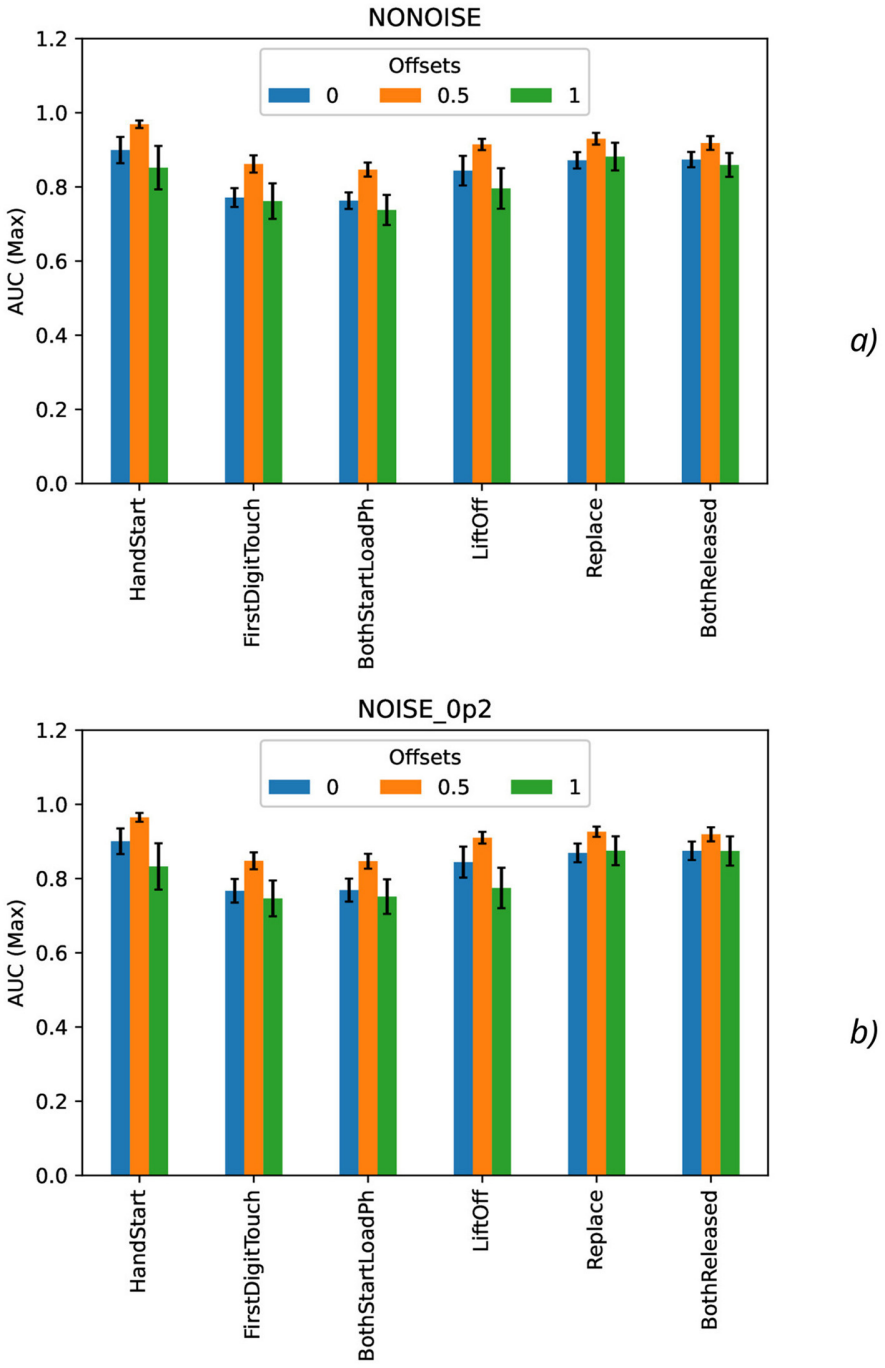
Comparison of maximal AUC values as a function of the number of samples for the different offset values (colors) without the synthetic noise **(A)** and with the synthetic noise σ_*synth*_ = 0.2 **(B)**. The error bars denote the standard deviation values.

**Figure 8:**
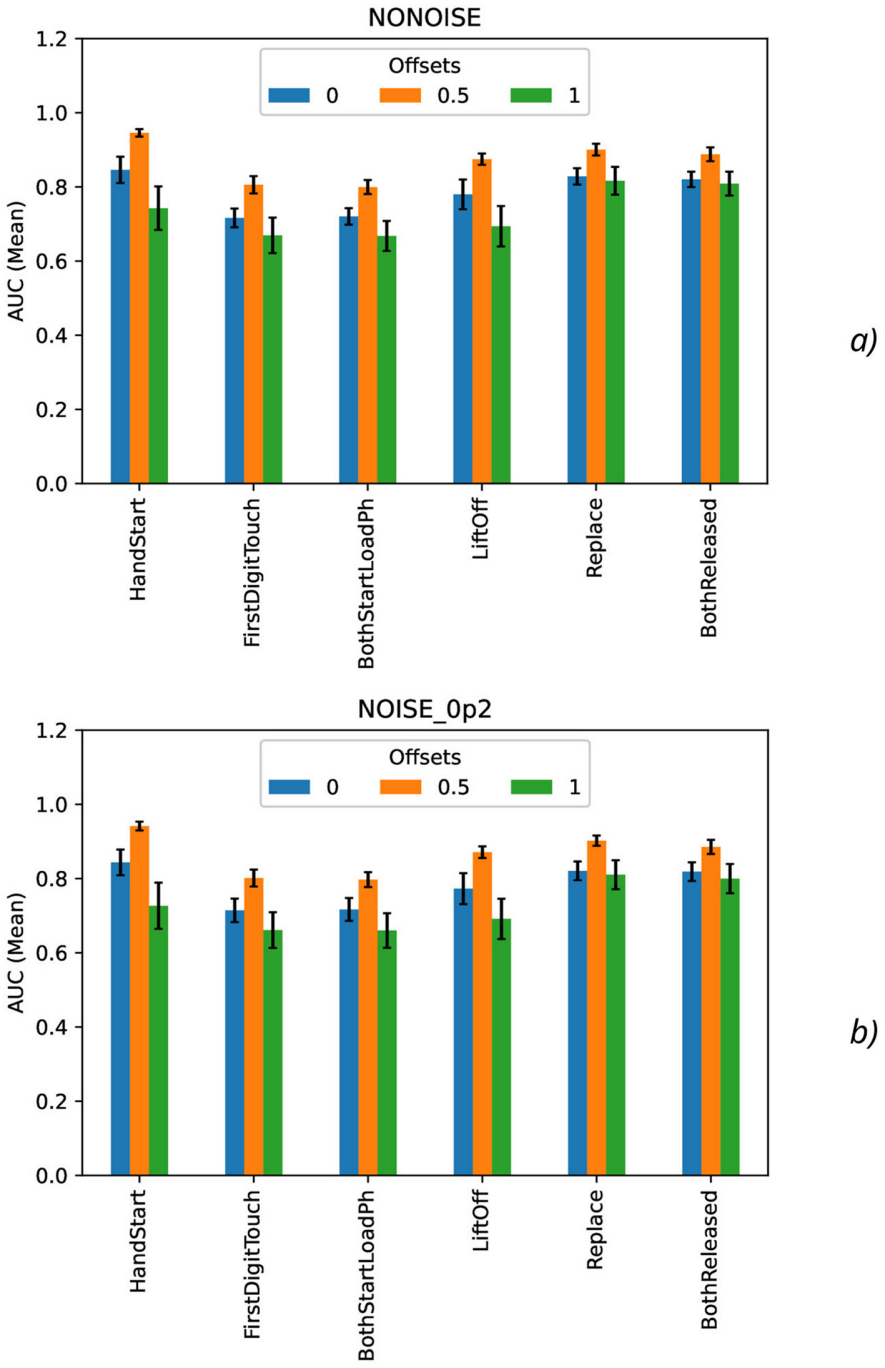
Comparison of mean AUC values as a function of the number of samples for the different offset values (colors) without the synthetic noise **(A)** and with the synthetic noise σ_*synth*_ = 0.2 **(B)**. The error bars denote the standard deviation values.

**Figure 9:**
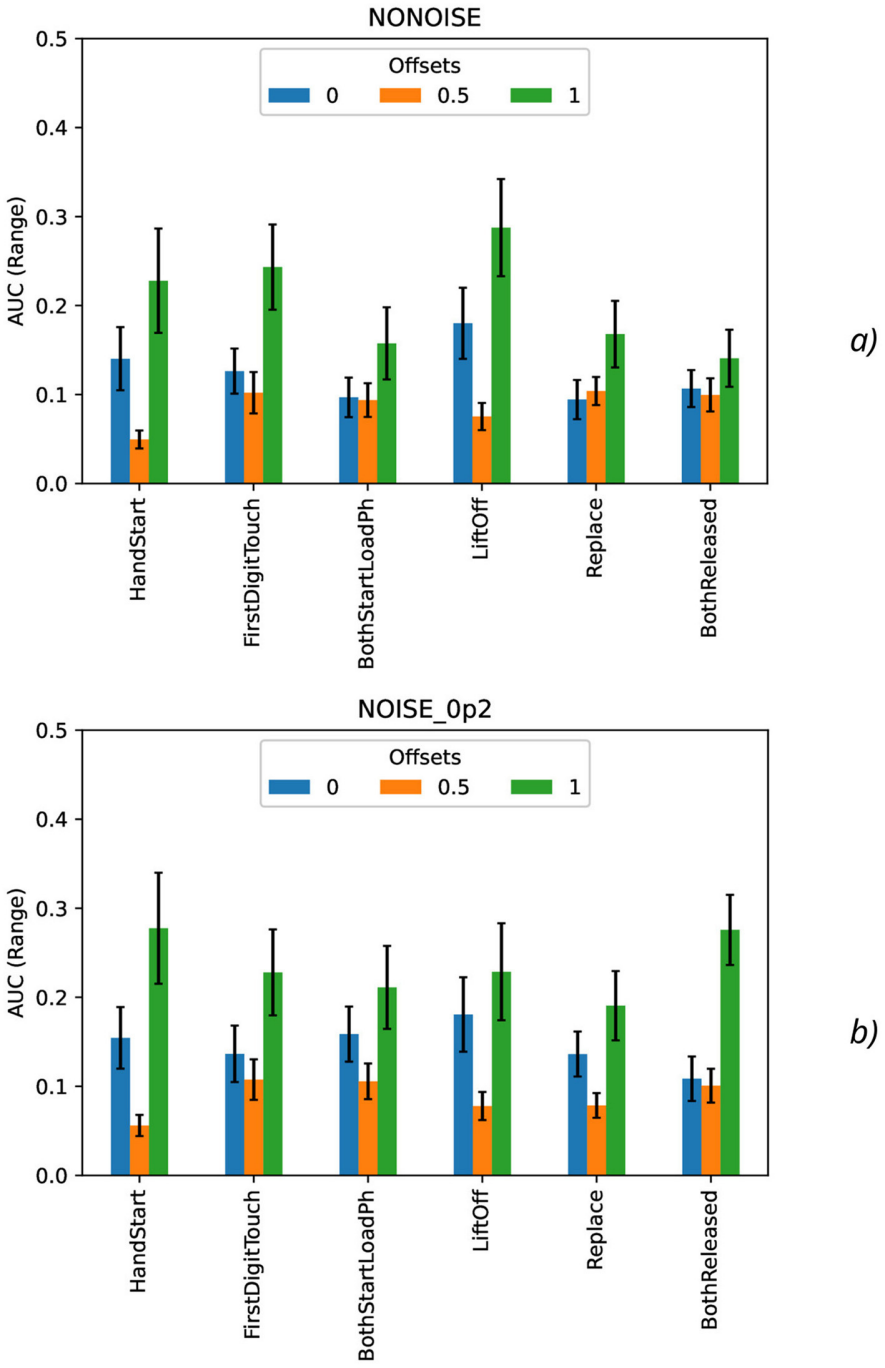
Comparison of range AUC values as a function of the number of samples for the different offset values (colors) without the synthetic noise **(A)** and with the synthetic noise σ_*synth*_ = 0.2 **(B)**. The error bars denote the standard deviation values.

**Figure 10:**
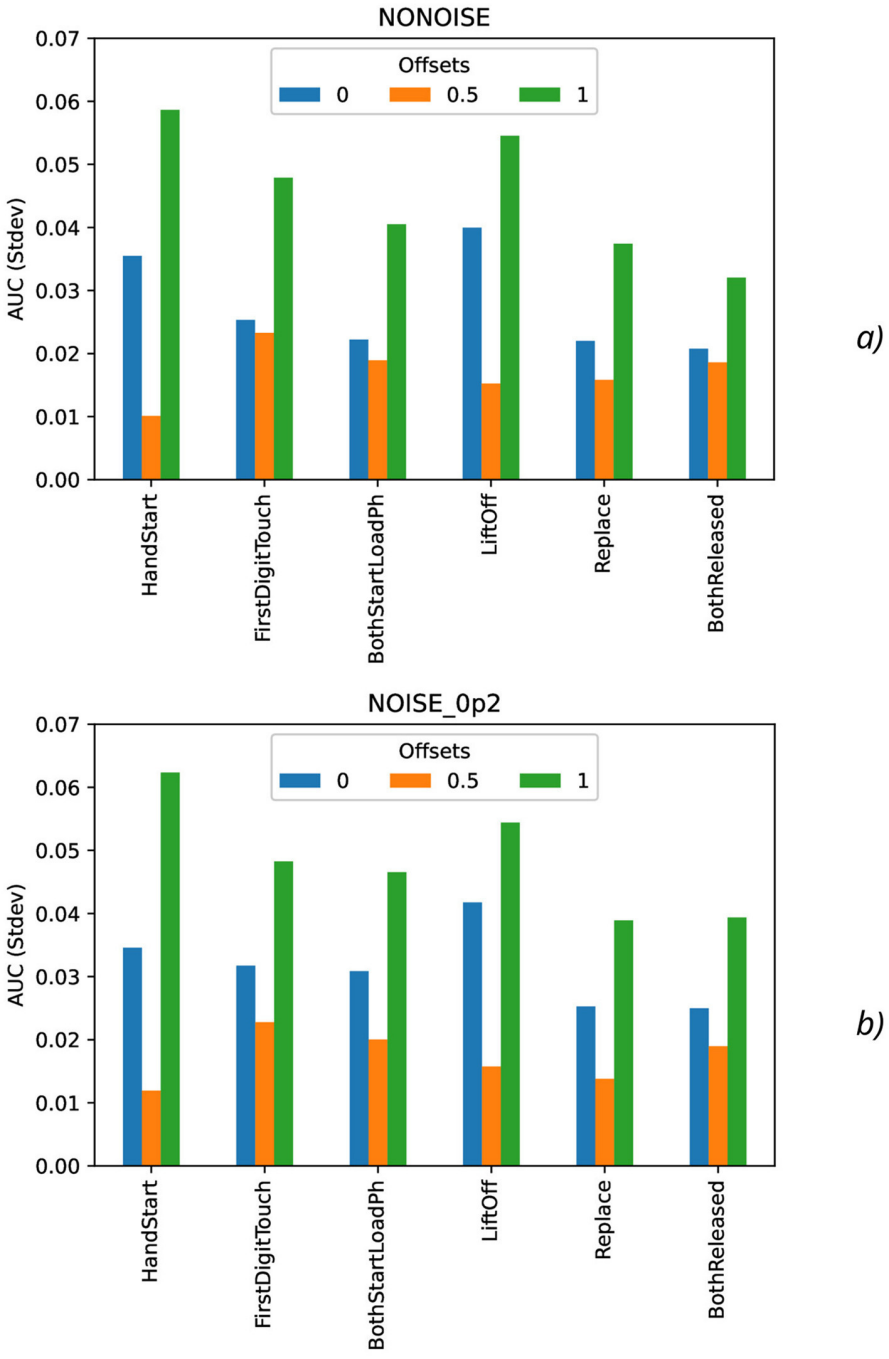
Comparison of standard deviation AUC values as a function of the number of samples for the different offset values (colors) without the synthetic noise **(A)** and with the synthetic noise σ_*synth*_ = 0.2 **(B)**.

Calculation of maximal (Figure 7), mean (Figure 8), minimal, and range (Figure 9), and standard deviation (Figure 10) of AUC values was performed for the different offset values and synthetic noise σ_*synth*_ values.

From the results obtained, the similar tendency can be observed for all actions: maximal AUC values (Figure 7) are higher for offset = 0.5 than for other offset values (0 and 1) and significantly bigger than standard deviation values. The maximal AUC values for offset = 0 are slightly higher than for offset = 1, but these differences cannot be considered as statistically significant and they are in the limits of standard deviation values.

The mean AUC values (Figure 8) are even more higher for offset = 0.5 than for other offset values (0 and 1) and significantly bigger than standard deviation values also. The mean AUC values for offset = 0 are even more higher than for offset = 1, but again these differences cannot be considered as statistically significant and they are in the limits of standard deviation values.

On the contrary, the range AUC values (Figure 9), which are differences between maximal and minimal AUC values, are lower for offset = 0.5 than for other offset values (0 and 1), and these differences are significantly bigger than standard deviation values. Similarly, the range AUC values for offset = 0 are lower than for offset = 1, and these differences are also statistically significant and beyond the limits of standard deviation values.

As it was seen from the previous Figure 9, the standard deviation AUC values (Figure 9) are significantly lower for offset = 0.5 than for other offset values (0 and 1). Similarly, the standard deviation AUC values for offset = 0 are significantly lower than for offset = 1.

To analyze the metrics for the steady region for *N* in the range from 1,000 to 10,000 samples, AUC (mean ± stdev) values were calculated (Table 2) along with the other metrics such as maximal AUC values and ranges (differences between maximal and minimal AUC values) (Table 3). The bold font in Table 3 denotes the highest values for the same level of σ_*synth*_, and the italic font does the lowest ones. HandStart action demonstrates the highest AUC values, and FirstDigitTouch and BothStartLoadPhase demonstrates the lowest ones. The important aspect is that for different actions, implication of the natural noise (presented by offsets) leads to the different consequences. For example, Replace and BothReleased actions have the lowest AUC decrease and highest AUC values for offset 0. As a possible explanation for these results, Replace and BothReleased actions can have the higher PAS-NR (in comparison with other actions) for offset 0 because of the more EEG activity for “after” (post-process) part of the relevant sampling. At the same time, mediocre performance for FirstDigitTouch and BothStartLoadPhase can be explained by their coincidence in time (see Figure 1) that is the real drawback of the GAL dataset used.

**Table 2:** AUC (mean ± stdev) (Figures 8, 10) values for the steady region (Figure 4) from 1,000 to 10,000 samples.

	**offset** = **0**			
Noise (σ_*synth*_)	0	0.01	0.1	0.2
HandStart	**0.845** ± 0.036	**0.845** ± 0.033	**0.850** ± 0.034	**0.844** ± 0.035
FirstDigitTouch	*0.716* ± 0.025	*0.719* ± 0.029	*0.719* ± 0.033	*0.714* ± 0.032
BothStartLoadPhase	*0.720* ± 0.022	*0.720* ± 0.026	*0.719* ± 0.029	*0.717* ± 0.031
LiftOff	0.780 ± 0.040	0.781 ± 0.038	0.778 ± 0.044	0.773 ± 0.042
Replace	0.828 ± 0.022	0.824 ± 0.023	0.827 ± 0.019	0.821 ± 0.025
BothReleased	0.820 ± 0.021	0.820 ± 0.022	0.819 ± 0.026	0.819 ± 0.025
	**offset** = **0.5**			
Noise (σ_*synth*_)	0	0.01	0.1	0.2
HandStart	**0.946** ± 0.011	**0.945** ± 0.011	**0.945** ± 0.013	**0.942** ± 0.012
FirstDigitTouch	*0.806* ± 0.023	*0.804* ± 0.023	*0.802* ± 0.022	*0.801* ± 0.023
BothStartLoadPhase	*0.800* ± 0.019	*0.801* ± 0.019	*0.801* ± 0.019	*0.797* ± 0.020
LiftOff	0.875 ± 0.015	0.875 ± 0.016	0.878 ± 0.016	0.871 ± 0.016
Replace	0.900 ± 0.016	0.902 ± 0.014	0.900 ± 0.016	0.902 ± 0.014
BothReleased	0.888 ± 0.019	0.888 ± 0.018	0.886 ± 0.016	0.885 ± 0.019
	**offset** = **1**			
Noise (σ_*synth*_)	0	0.01	0.1	0.2
HandStart	0.743 ± 0.059	0.741 ± 0.062	0.736 ± 0.062	0.727 ± 0.062
FirstDigitTouch	*0.669* ± 0.048	*0.673* ± 0.042	*0.671* ± 0.046	*0.661* ± 0.048
BothStartLoadPhase	*0.668* ± 0.041	*0.668* ± 0.043	*0.670* ± 0.040	*0.660* ± 0.047
LiftOff	0.694 ± 0.055	0.700 ± 0.051	0.698 ± 0.060	0.691 ± 0.054
Replace	**0.816** ± 0.037	**0.822** ± 0.037	**0.813** ± 0.038	**0.810** ± 0.039
BothReleased	**0.809** ± 0.032	**0.806** ± 0.035	**0.805** ± 0.032	**0.800** ± 0.039

The bold font denotes the highest values for the same level of the noise, and the italic font does the lowest ones.

**Table 3:** Maximal and range AUC values for the steady region from 1,000 to 10,000 samples (Figures 4, 11, 12).

	**offset = 0**							

	AUC (max)				AUC (range)			
Noise (σ_*synth*_)	0	0.01	0.1	0.2	0	0.01	0.1	0.2
HandStart	**0.899**	**0.909**	**0.908**	**0.901**	0.140	0.146	0.150	0.154
FirstDigitTouch	0.771	0.787	0.795	0.767	0.126	0.131	0.143	0.137
BothStartLoadPh	0.763	0.767	0.781	0.769	0.097	0.115	0.126	0.159
LiftOff	0.844	0.861	0.848	0.844	**0.180**	**0.186**	**0.245**	**0.181**
Replace	0.872	0.873	0.867	0.869	0.094	0.101	0.094	0.136
BothReleased	0.874	0.876	0.871	0.875	0.107	0.112	0.132	0.109
	**offset** = **0.5**							
	AUC (max)				AUC (range)			
Noise (σ_*synth*_)	0	0.01	0.1	0.2	0	0.01	0.1	0.2
HandStart	**0.969**	**0.967**	**0.972**	**0.965**	0.050	0.054	0.058	0.056
FirstDigitTouch	0.862	0.855	0.850	0.848	**0.102**	**0.122**	**0.100**	**0.108**
BothStartLoadPh	0.847	0.842	0.839	0.847	0.094	0.082	0.087	**0.106**
LiftOff	0.915	0.913	0.916	0.910	0.075	0.076	0.086	0.078
Replace	0.930	0.932	0.932	0.926	**0.104**	0.080	0.091	0.079
BothReleased	0.918	0.914	0.914	0.919	0.100	0.092	0.076	0.101
	**offset** = **1**							
Noise (σ_*synth*_)	0	0.01	0.1	0.2	0	0.01	0.1	0.2
HandStart	0.852	0.842	0.841	0.833	0.228	0.238	0.249	**0.278**
FirstDigitTouch	0.762	0.758	0.749	0.747	0.243	0.183	0.193	0.228
BothStartLoadPh	0.738	0.749	0.740	0.751	0.158	0.221	0.223	0.211
LiftOff	0.796	0.809	0.791	0.775	**0.288**	**0.289**	**0.261**	0.229
Replace	**0.882**	**0.886**	**0.883**	**0.875**	0.168	0.158	0.167	0.191
BothReleased	0.859	0.870	0.853	0.874	0.141	0.168	0.143	**0.276**

The bold font denotes the highest values for the same level of the noise, and the italic font does the lowest ones.

### 3.3 Detrended fluctuation analysis

Detrended Fluctuation Analysis (DFA) (Peng et al., 1993, 1995; Bianchi, 2020) was applied to analyze fluctuations of AUC values and the correspondent Hurst exponent values after eliminating the temporal trend (**Figure 13**). For the time sequences, the Hurst exponent value (*H*) can indicate whether a process is persistent or anti-persistent, but here the Hurst exponent is used for the other purpose, namely, for quantitative estimation of fluctuations svariability.

In general, the Hurst exponent, *H*, is intrinsically related to the fractal dimension, which quantifies the “roughness” or variability of a time series (Hurst, 1956). Specifically, the value of *H* provides insight into the degree of smoothness in the data: Sequences that exhibit greater variability and are more irregular (i.e., more jagged) are associated with lower values of *H*, approaching zero. Conversely, smoother sequences yield values of *H* closer to one. This relationship between *H* and the fractal dimension is instrumental in characterizing the long-term dependence and self-similarity in stochastic processes. The Hurst exponent can also characterize a process (Bianchi, 2020) depending on the range of the measured values: *H* in the range [0.0, 0.5) corresponds to a very noisy process; the value *H* = 0.5 relates to uncorrelated process; *H* in the range (0.5, 1.0] relates to persistency where long-range correlations and relatively little noise can be observed; and *H*>1.0 characterizes a non-stationary process with stronger long-range correlations. The correspondent open-source Python package “fathon” was used for DFA and further analysis of metric fluctuations (Bianchi, 2020).

## 4 Discussion

The results obtained show that different actions can be classified with the quite different reliability. The different kinds of physical activity take the different level of physical activation and the correspondent EEG activity, for example, HandStart (fingers, palm, forearm, and shoulder are activated) includes involvement of more limbs than LiftOff (fingers, palm, and forearm) and Replace (fingers, palm, and forearm), and even more than BothReleased (several fingers and palm), BothStartLoadPhase (two fingers), and FirstDigitTouch (one finger). It should be noted that the observed performance of classification demonstrates some correlation where the higher performance by AUC (Figure 4) corresponds to the more pronounced physical activity in the following order from the highest AUC values to the lowest ones: HandStart → LiftOff → Replace → BothReleased → BothStartLoadPhase → FirstDigitTouch (Figure 4).

In addition, for *N* values in the range [100, 1,500], HandStart action demonstrates the asymmetric behavior with regard to the offset values 0 and 1 (Figure 4C), namely: AUC values grows much faster with *N* for offset = 1 (the dotted and smoothed red lines in Figure 4A) than for offset = 0 (the dotted and smoothed red lines in Figure 4C). It means that the related brain activity measured as “before” (pre-process) part of the correspondent EEG time sequences is more pronounced than “after” (post-process) part. As a result, this phenomenon allows us to classify HandStart before the actual physical action even as it was assumed in our previous studies (Gordienko et al., 2021c,b; Kostiukevych et al., 2021). It is in contrary to the kinds of activities that demonstrate similar behavior: similar growth of AUC values for *N* values in the range [100, 1,500] the offset values 0 and 1, and decay for *N*>1500.

In general, AUC values are higher for the offset value 0.5 (in comparison with the offset values 0 and 1), steadily for *N* values in the range [100, 1,500] and nearly constant for *N*>1, 500 for all kinds of activities (Figure 4B) as it was also shown in our previous studies (Gordienko et al., 2021c; Kostiukevych et al., 2021). That is why labeling by the offset of 0.5 seems to be the more significant for the classification problem. It should be noted that the uncertainty of AUC values estimated as their standard deviations decreases with an increase of *N* up to *N* = 2, 000 for offset = 0.5 and up to *N* = 3, 000 for offset = 0 and offset = 1. It should be noted the visually very pronounced fluctuations of all these metrics with *N* can be explained by the influence of the non-relevant (to the current physical activity) regions of the increased time sequence under investigation.

It should be noted that application of the natural NDA by increasing *N* leads to the better micro and macro AUC values for *N* values beyond the physical action duration which is ~0.3 s (that is equal to ~*N*=150 samples, see Figure 1) and up to ~3 s (*N* = 1,500) for offset = 0.5. For example, micro and macro AUC values are equal to ~0.65 for sampling length *N* = 200 samples (that corresponds to ~0.4 s), and increase of *N* up to *N* = 1,500 leads to the better micro and macro AUC values equal to ~0.87 (Figures 5, 6). But to the moment it is unclear whether this improvement caused by the availability of EEG signals relevant to the physical action beyond action itself or by natural NDA. The additional interesting aspect is that micro and macro AUC values are much lower for the offset 0 and 1 (in comparison with offset = 0.5), but AUC values are improving with *N* (Figures 5, 6) up to ~6–7 s (*N* = 3,000) for offset = 0 and offset = 1. It means that heavy bias of labeling is not useful because it leads to distortion of PAS-NR due to the lower signal and higher noise values. Application of the synthetic NDA (Figures 5, 6) in the wide range of noise levels (σ_*synth*_ from 0.001 up to 0.2) demonstrates the general stability of the DNN used for classification of all activities with the similar micro and macro AUC values in the limits of their fluctuations.

**Figure 11:**
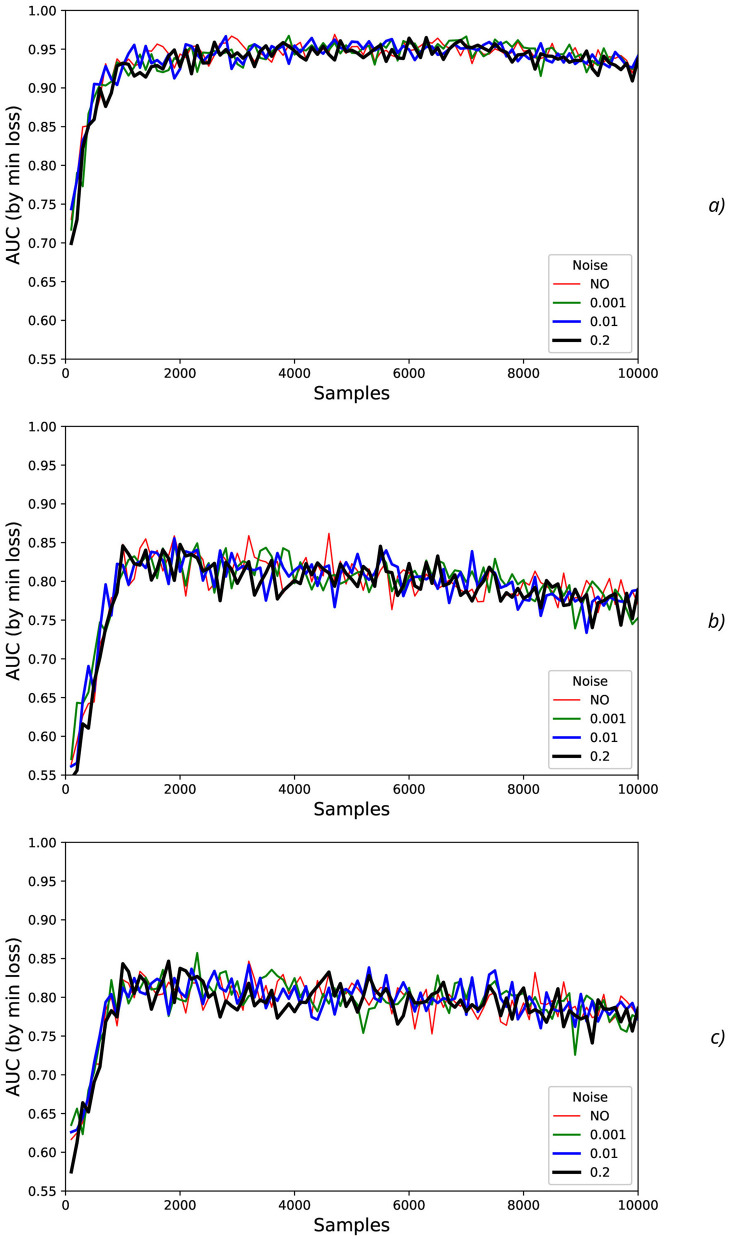
Noise influence on AUC values for offset = 0.5: **(A)** HandStart, **(B)** FirstDigitTouch, and **(C)** BothStartLoadPhase.

**Figure 12:**
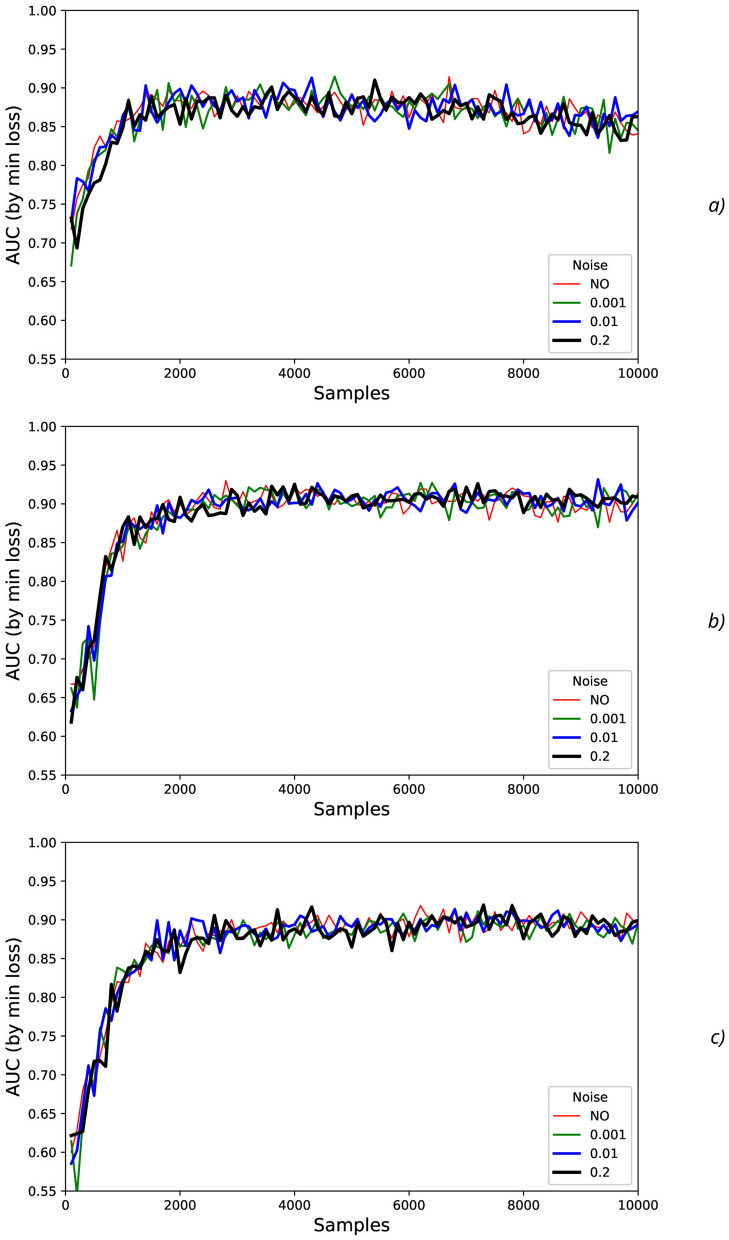
Noise influence on AUC values for offset=0.5 (continued from Figure 11): **(A)** LiftOff, **(B)** replace, **(C)** BothReleased.

AUC fluctuations caused by the added synthetic NDA, shown in Figures 11, 12, are not significant in comparison with AUC fluctuations without synthetic NDA due to increase of sampling size *N*.

**Figure 13:**
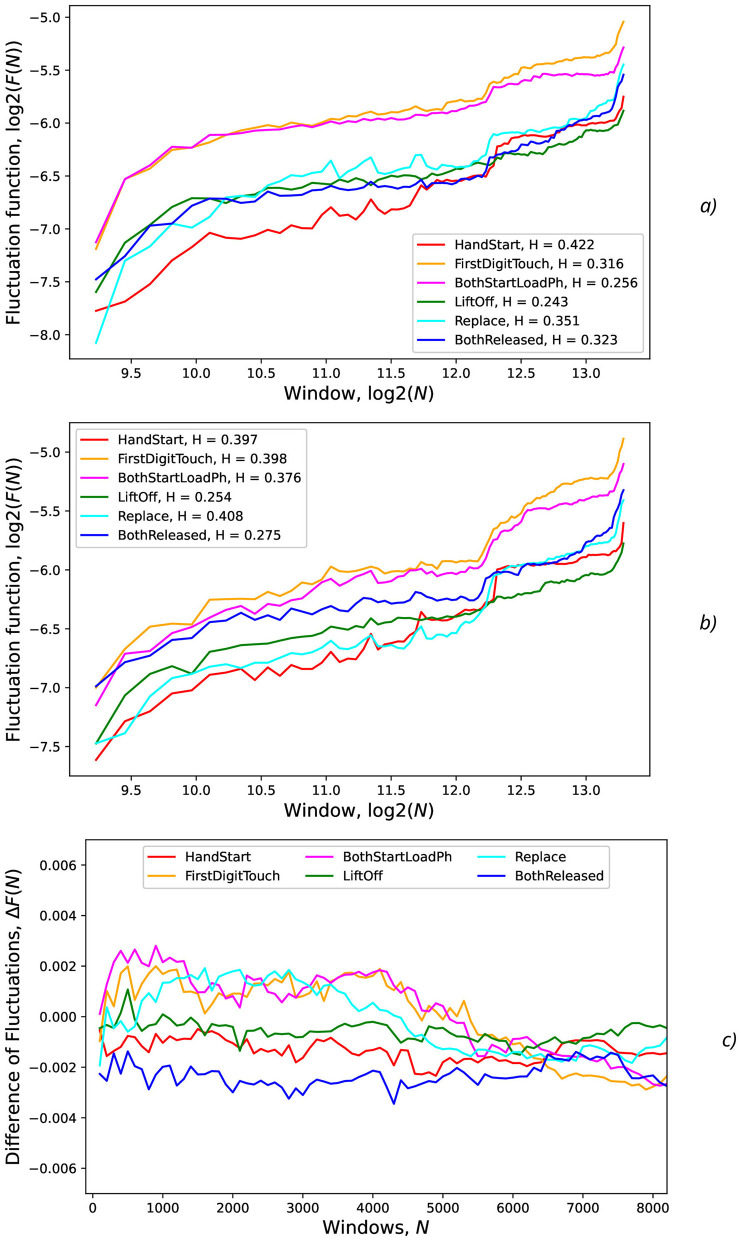
Fluctuations vs. the number of samples (*N*) in the input for various actions and levels (standard deviations) of the synthetic noise: **(A)** σ_*synth*_ = 0 (no noise), **(B)** σ_*synth*_ = 0.2, **(C)** difference of fluctuations from previous regimes without and with noise. The legends contain Hurst exponent values.

To characterize AUC fluctuations (Figures 11, 12) with regard to the added synthetic NDA, the DFA was applied and analyzed for original (non-added noise) EEG time sequences (Figure 13A) and ones with NDA (Figure 13B). From DFA point of view, some very intensive actions (such as HandStart and LiftOff) demonstrate the very high stability to noise data augmentation with negligible changes of fluctuation amplitudes measured like differences (Figure 13) between the correspondent AUC fluctuation values for original (without added noise) (Figure 13A) and noise-augmented EEG time sequences (Figure 13B).

For FirstDigitTouch, BothStartLoadPhase, and Replace activities, the synthetic NDA actually lead to decrease of the AUC fluctuations (Figure 13C) with slow decay of this improvement with increase of *N* (due to above mentioned non-relevant noisy neighboring regions, i.e., the natural NDA). In contrary, for HandStart and BothReleased activities, the synthetic NDA actually lead to increase of the AUC fluctuations (Figure 13C) with slow decay also. Liftoff activity does not demonstrate any significant changes.

The measurements of the Hurst exponent values *H*_*full*_ (the full range of window scales with *n* < 10, 000, time < 20 s), *H*_*low*_ (the low window scales with *n* < 1, 000, time < 2 s), and *H*_*high*_ (the bigger window scales with *n*>1, 000, time >2 s) were performed for various actions and levels (standard deviations σ_*synth*_ = 0) of the synthetic noise (Table 4). *H* values are rounded to 2 decimal digits in Table 4 because the bigger number of significant digits seems to be statistically insignificant.

**Table 4:** Hurst exponents *H*_*full*_, *H*_*low*_, and *H*_*high*_ (rounded to 2 decimal digits) for various actions and levels (by standard deviations σ_*synth*_) of the synthetic noise.

	σ_*****synth*****_ = 0 **(no noise)**			σ_*****synth*****_ = 0.2 **(noise)**		
**Hurst exponent**	***H*_*full*_**	***H*_*low*_**	***H*_*high*_**	***H*_*full*_**	***H*_*low*_**	***H*_*high*_**
HandStart	0.42	0.78	0.43	0.40	0.67	0.41
FirstDigitTouch	0.32	0.92	0.29	0.40	0.64	0.40
BothStartLoadPhase	0.26	0.92	0.23	0.38	0.67	0.37
LiftOff	0.24	0.87	0.21	0.25	0.67	0.22
Replace	0.35	1.07	0.28	0.41	0.72	0.42
BothReleased	0.32	0.77	0.30	0.28	0.50	0.24

The general tendency is that for the low window scales (*n* < 1, 000, time < 2 s), *H*_*low*_ values are higher in comparison with *H*_*high*_ values for the bigger window scales (*n*>1, 000, time >2 s) that can be seen from the slope of curves in Figure 13 and Table 4. It means that EEG fragments with the duration of scale *n* < 1, 000 (time < 2 s) demonstrate the scaling behavior of the higher complexity than the fragments *n*>1, 000 (time >2 s), i.e., *H*_*low*_>*H*_*high*_ (Table 4). It should be noted that step-like increases in the middle and in the end of all curves in Figure 13 can be explained by overlapping with the next portion of PAS data related to the other trial of recorded physical activities which are contained in the whole timeline of the experimental EEG data.

Despite the previously mentioned findings, the study has several limitations that should be taken into account in future works. First, the use of a single epoch for training (which was observed to be enough for saturation of the training process of the relatively small DNN with the quite small capacity) may limit the model's overall performance. In future research, the multiple training epochs for the more complex DNN should be employed to potentially improve model performance and generalization with attention to the impact of hyperparameter tuning (e.g., learning rate, batch size) on model performance and convergence. Second, the focus on feasibility analysis rather than maximizing performance might have constrained the exploration of more complex DNN architectures. In the next stage of the investigation, the more complex DNN architectures (such as deeper CNNs, recurrent neural networks, and transformer models) should be performed with a more comprehensive hyperparameter search to optimize model performance to potentially achieve higher classification accuracy. Third, the study relies mainly on the GAL dataset, which may not fully capture the variability and complexity of real–world EEG signals. In the extended version, this study should include investigation of the model's performance on other publicly available EEG datasets with different characteristics in the other controlled and realistic environment to improve the generalizability of the findings. Fourth, the analysis is limited to a specific set of NDA techniques, and the impact of other noise sources or more sophisticated DA methods should be also explored. Moreover, the impact of other NDA techniques (mentioned in the introductory part of the study, such as generative training and others) will be necessary to improve model robustness and explore the impact of physiological noise (e.g., muscle artifacts, eye blinks) and environmental noise on model performance. In addition, assessing the additional metrics particularly with regard to Structural Similarity Index (SSIM) and Peak Signal-to-Noise Ratio (PSNR) in future research stages will be highly intriguing and valuable. SSIM could provide insights into the structural similarity between original and noise-augmented EEG signals, helping evaluate how natural noise preserves critical signal features. Meanwhile, PSNR could serve as a measure of distortion, indicating how much the augmented signals deviate from the original ones, which is crucial for maintaining signal integrity in classification tasks.

## 5 Conclusion

This research contributes to the field of EEG-based BCI by investigating the impact of different types of noise on the classification of physical activities by the following main novel aspects and contributions: systematic investigation of natural noise, quantitative analysis of noise impact, and analysis of offset effects. The study introduces the concept of “natural noise” by considering EEG data from neighboring regions, simulating real-world scenarios with varying levels of background EEG activity. The researchers utilize metrics such as AUC and DFA to quantitatively assess the impact of both natural and synthetic noise on classification performance, providing valuable insights into the model's robustness. By analyzing the impact of different label offsets (0, 0.5, 1), the study provides valuable insights into the optimal time window for EEG signal analysis and classification. These novel aspects contribute to a better understanding of the challenges and limitations of EEG-based BCI systems in real-world scenarios and provide valuable guidance for future research in this area.

The following key aspects of the methodology contribute to achieving the goal: DA by natural NDA and synthetic NDA, varying NDA parameters including input sequence length, and thorough performance evaluation including DFA Analysis. The introduction of both natural and synthetic noise during DA helps the model to become more robust and generalize better to real-world scenarios with varying levels of noise. As to the natural NDA by including EEG data from neighboring regions, the model learns to handle variations in EEG signals due to temporal shifts and contextual influences. As to the synthetic NDA, adding Gaussian noise increases the model's tolerance to random fluctuations and noise in the EEG data. The use of input sequences with varying lengths (N) allows the model to assess its performance under different levels of “natural noise” introduced by the inclusion of irrelevant EEG data. This helps to understand how the model's performance is affected by the amount of surrounding EEG data. For performance evaluation, the use of multiple metrics, including AUC (micro and macro), accuracy, and loss, provides a comprehensive evaluation of the model's performance. For DFA, analysis helps to quantify the variability and complexity of the AUC fluctuations, providing insights into the model's behavior under different noise conditions. By incorporating these techniques, the authors aim to understand the feasibility and limitations of classifying EEG signals related to physical activities in the presence of noise, which is crucial for the practical application of BCI systems in real-world settings.

The results obtained allow us to conclude that the relatively simple DNN with components of FCN and CNN even can be effectively used to classify physical activities (namely, hand manipulations) from the GAL dataset. Application of natural and synthetic noises imitates the possible influence from environment. It should be noted that synthetic noise influence (due to Gaussian NDA with higher σ values) has the lower impact on the general ability to provide the better reliable classification of physical activities than natural noise influence (due to increase of the sampling size *N*) that can significantly improve the performance with reaching the stable metric values after some noise increase.

AUC fluctuations caused by the added synthetic NDA are not significant in comparison with AUC fluctuations without synthetic NDA due to increase of sampling size *N*. It should be emphasized that application of the natural NDA by increasing *N* leads to the better micro and macro AUC values for *N* values beyond the action duration which is ~0.3 s and up to ~3 s (*N* = 1,500) for offset = 0.5. But to the moment the open question is whether this improvement caused by the availability of EEG signals relevant to the physical action beyond action itself or by natural NDA. This aspect should be resolved by further investigations and on other open EEG datasets.

Application of the synthetic NDA in the wide range of noise levels (σ_*synth*_ from 0.001 up to 0.2) demonstrates the general stability of the DNN used for classification of all activities with the similar micro and macro AUC values in the limits of their fluctuations.

DFA allows us to investigate the fluctuation properties and calculate the correspondent Hurst exponents for the quantitative characterization of their variability. As a result of this research, some PAs can be divided in separate groups of actions that can be characterized by complexity and the feasibility of their classification: the easiest (HandStart), medium (LiftOff, Replace, and BothReleased), and hardest (BothStartLoadPhase and FirstDigitTouch) classification.

A general trend is observed in the behavior of the Hurst exponent *H* across varying time window scales in EEG data. Specifically, for shorter time window scales (i.e., < 2 s), the values of *H*_*low*_ tend to be significantly higher than those for longer time window scales (i.e., >2 s), denoted as *H*_*high*_. This suggests that EEG segments with durations shorter than 2 s exhibit greater scaling complexity than those of longer durations. In particular, *H*_*low*_ can exceed *H*_*high*_ by a factor of 2 to 3 during certain physical actions, indicating a marked increase in complexity for these shorter time-scale fragments.

In general, this approach of adding natural noise by extending sampling size for small DNNs can be used during porting such models to Edge Computing infrastructures on devices with the very limited computational resources because the statistically reliable results were obtained by the relatively small DNN with the low resource requirements (Kochura et al., 2019; Gordienko et al., 2020, 2021a). The additional possible improvement can be obtained due to analysis of the optimal configuration for training and inference stages of the whole workflow that is especially important for distributed infrastructures (Kochura et al., 2017b; Taran et al., 2017; Gordienko et al., 2021a; Kochura et al., 2017a). Similar research could be also useful for classification of GAL-like and any other PAs before their actual start when some prediction with PA classification can be performed on the EEG activity before PA even. By this approach, the human EEG activity can be estimated with some proactive feedback such as continuation of PAs which were initiated by brain only, but unfortunately the PAs were not continued due to fatigue or some limited physical abilities, but the future detailed investigation should be performed to take into account the more various kinds of PAs.

## Data Availability

The original contributions presented in the study are included in the article/supplementary material, further inquiries can be directed to the corresponding author.
